# Thoracic Vertebra Chance Fracture Resulting from Mechanical CPR

**DOI:** 10.5334/jbsr.3564

**Published:** 2024-04-08

**Authors:** Thomas Saliba, Sanjiva Pather, Olivier Cappeliez

**Affiliations:** 1 Hopital de Braine L’Alleud, Braine L’Alleud, Belgium; 2 Hopital de Braine L’Alleud, Braine L’Alleud, Belgium; 3 Hopital de Braine L’Alleud, Braine L’Alleud, Belgium

**Keywords:** Chance, fracture, spine, CPR, resuscitation

## Abstract

Chest compressions, used in cardiopulmonary resuscitation (CPR), cause rib and sternum fractures in around 79% and 54% of patients, respectively. Spinal fractures resulting from CPR are far rarer. We present the case of a 70-year-old man who underwent mechanical CPR after choking whilst eating. The patient received a cerebral and thoracic CT scan upon arrival to the hospital. The cerebral scan was normal, but the chest CT scan revealed signs of ankylosing spondylitis and an unstable Chance fracture of the 12th thoracic vertebra. The patient was hospitalised but passed away. This case highlights the need for awareness of uncommon spine fractures due to the high associated morbidity.

*Teaching point:* In patients who have undergone thoracic compressions, one should not only search for rib fractures but also for spine fractures, which, though uncommon, have a far greater impact on the patient’s morbidity, especially in patients with predisposing spine conditions.

## Introduction

Chest compressions have been part of the cardiopulmonary resuscitation (CPR) algorithm since 1960, having undoubtedly contributed to saving many lives [1, 2]. Rib fractures are, however, a common complication resulting from CPR, with studies reporting between 26% and 80% of patients suffering from over six or more costal fractures [3]. In contrast, spine fractures are far rarer and subsequently less reported in the literature [2]. We present the case of a 70-year-old man who received mechanical CPR, resulting in a Chance fracture.

## Case Report

**Figure 1:**
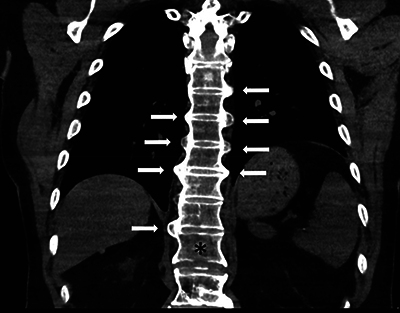
Coronal CT scan image showing the extensive syndesmophytes (arrows) along the thoracic spine and the fractured vertebra (black star).

**Figure 2:**
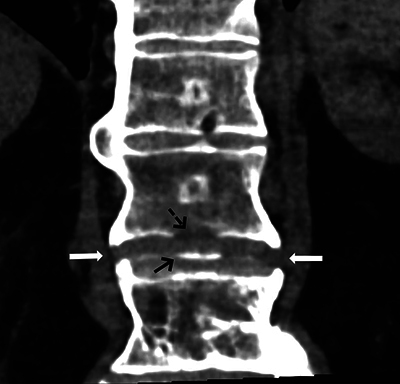
Coronal CT-scan image showing the fractured syndesmophytes (arrows) alongside a partial avulsion of the inferior endplate (black arrow) of T12 corresponding to the cortical defect (dotted arrow).

**Figure 3:**
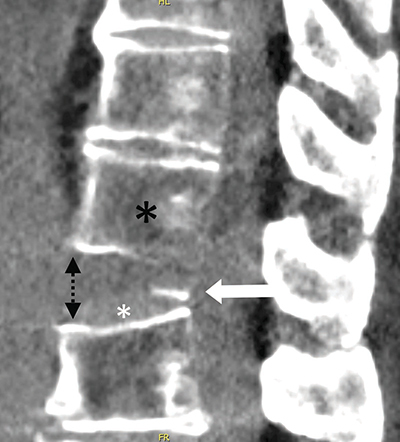
Sagittal CT-scan image showing a partial avulsion of the inferior endplate (arrow) of T12 (black star), which has remained attached to L1, along with the slightly hyperdense disc (white star). There is a diastasis between the anterior parts of the vertebral bodies (black dotted arrows) compared to the posterior part.

A 70-year-old man suffered a cardiac arrest after choking on food whilst in a restaurant and received mechanical CPR on location with a LUCAS device (Physio-Control Inc.; Lund, Sweden) for 25 minutes. The patient was resuscitated and transported to the hospital, where a cerebral and thoracic CT scan were performed as the patient remained unconscious and was neurologically unexaminable. The cerebral CT scan was normal. The thoracic CT scan showed multiple bilateral rib fractures as well as a bamboo spine appearance compatible with ankylosing spondylitis (Figure 1). Further examination revealed a slightly displaced, unstable, Chance fracture of the 12th thoracic vertebra (Figures 2 and 3). The patient was immobilised and transferred to the intensive care unit, where he passed away 15 days later after failing to regain consciousness following prolonged cerebral hypoxia.

## Discussion

Chest compressions are an essential part of CPR, with rib fractures being a common complication [3]. Vertebral fractures resulting from CPR, however, are far rarer, with most being discovered post-mortem [4]. In a series of 705 autopsy cases, only one patient had a cervical spine fracture [5]. Of the currently known cases, the majority resulted in instances where mechanical compression was used [4, 6]. Interestingly, mechanical compression devices have also been linked with skeletal, soft tissue and cardiac injuries [4]. Mechanical CPR is responsible for more rib fractures (79% vs. 65%) as well as sternal fractures (58% vs. 54%) compared to manual CPR [7]. In cases of vertebral fractures related to CPR, the pattern observed is more typical of cases of extension injuries, rather than the axial loading patterns seen in trauma [4]. Risk factors for CPR-related fractures are thought to include osteopenia, osteoporosis and kyphosis, though our patient did not have any of these [4, 8]. Our patient did, however, have signs of ankylosing spondylitis, which causes patients to have a reported higher odds ratio of 7.7 for developing spine fractures compared to the average population [9].

## Conclusion

Multiple rib fractures are a typical complication in patients who have received CPR, occurring in up to 79%. Spine fractures resulting from CPR are far rarer, with the exact incidence being unknown, though possibly higher in patients having undergone mechanical chest compressions. We reported, what to our knowledge is, the first case of a patient with ankylosing spondylitis who suffered a Chance fracture following mechanical CPR. This case highlights the need to remain vigilant for CPR-related spine fractures, which, though infrequent, have a great impact on the patient’s morbidity.
